# Tipping the Fencesitters—The Impact of a Minimal Intervention Enhanced with Biological Facts on Swiss Student Teachers’ Perception of HPV Vaccination Safety

**DOI:** 10.3390/vaccines10020175

**Published:** 2022-01-23

**Authors:** Alla Keselman, Albert Zeyer

**Affiliations:** 1 Office of Engagement and Training, National Library of Medicine, National Institutes of Health, Bethesda, MD 20894, USA; 2 Institute for Education in Science and Social Studies, University of Education Lucerne, 6003 Lucerne, Switzerland; albert.zeyer@phlu.ch

**Keywords:** vaccine hesitancy, science for all, biology education, health education, science in daily life

## Abstract

Not much is known about the role of scientific knowledge in vaccination decision making. This study is based on previous findings that the concern about the human papillomavirus (HPV) agent mutating back to a virulent HPV was common among Swiss student teachers and turned out to be one factor of vaccine hesitancy. The study investigate the impact of a standard public health brochure describing the effectiveness, safety, and importance of HPV vaccination on young student teachers, and the additional effect of supplementing the standard brochure with biological arguments against the mutation concerns. It uses a pre-posttest design and assigns participants randomly to two groups, one reviewing a standard public health brochure, the other the same brochure enhanced with additional biological information. Participants in both groups showed a significant positive change in their beliefs about vaccination safety, effectiveness, and importance in preventing cervical cancer. Post hoc analysis showed significant safety beliefs gain for the subgroup of participants who received the biology-enhanced text and held moderate, rather than high or low, pretest safety beliefs—the so-called fencesitters. We conclude that these fencesitters may particularly profit from even minimal (biologically supplemented) interventions, an effect that should receive more attention in future research.

## 1. Introduction

Few studies have explored the effect of scientific knowledge on health reasoning and decision-making. As a consequence, we know little about the specific health situations in which scientific knowledge is beneficial, the desired content and depth of this knowledge, and the optimal ways to impart it.

The study described in this paper is a further step in a series of studies about vaccination hesitancy that has been conducted with Swiss schools, students, and pre-service teachers [1,2,3,4]. While addressing vaccine hesitancy has always been an important public health issue, the COVID-19 pandemic months amply demonstrated to the world the impact of vaccination refusal on societies. They also showed that the issue affects many countries and demographic groups. Young pre-service teachers are an important public for vaccination education because, on the one hand, they may have an important impact on students’ attitudes towards vaccination, while on the other hand, many of them will become parents themselves and thus will have to decide about vaccinations of their children [3].

The current study was prompted by the findings of two qualitative Swiss studies on HPV vaccination. The first of these studies [1] found that an important reason for HPV vaccination hesitancy in two Swiss secondary schools was the skepticism of both the mothers and teachers of students. In a second (follow-up) study, potential reasons for this skepticism were investigated with preservice teachers in a university of teacher education [4]. One major concern of these young female student teachers was the fear that the HPV vaccine in the human body could mutate into infectious particles that could then cause the very same cervical cancer the vaccine is meant to prevent.

To the best of our knowledge, so far, no studies have pointed out, not to mention investigated, biological concerns as reasons for vaccination hesitancy. Nevertheless, the two abovementioned studies suggest that biological information—in this case about the character of the vaccination particles as genetically engineered non-virulent markers that cannot mutate into viruses—could make a difference when it comes to the decision making of two key players for HPV vaccination in school. These two key players are the students’ teachers and mothers.

As far as we are aware, such a hypothesis has never been tested. Generally, the role of scientific knowledge in health decision making is unclear [5]. It is assumed that values play a more important role in these decisions than factual knowledge, even in experts’ and more so in laypersons’ decision making [6]. This may be the reason why no studies have looked at the effect of scientific knowledge on health decisions.

Given all these uncertainties, we opted for minimal intervention. This study explores if just integrating a paragraph about biological information on HPV vaccine particles may influence reported safety judgments in young student teachers. We gave the student teachers no introduction, no context, and no personal communication about the issue. The information was simply distributed, and the effect of this minimal intervention was measured. Actually, this is quite realistic, as it often is the only way how youths and their parents are informed about HPV and the HPV vaccination.

### 1.1. Human Papillomavirus (HPV): Epidemiology, Impact and Prevention

HPV is the most frequently occurring sexually transmitted infection [7]. While most cases of HPV resolve without negative health effects, some strains can cause cervical cancer, other types of cancer, and genital warts. Cervical cancer is the fourth most common cancer in women worldwide, accounting for 7.5% of all cancer deaths among women [8]. Approximately a quarter of these deaths occur in developed countries [8]. In Switzerland, where the data for this study were collected, each year approximately 5000 women are diagnosed with pre-cancerous cervical lesions that require invasive follow-up treatment and/or surgery [9].

First available in 2006, the HPV vaccination is a safe and effective preventative measure against cancers caused by HPV [10]. The vaccine is generally well tolerated and effective. Common side effects are minor, and severe allergic reactions are extremely rare [9]. The Swiss Federal Office of Public Health estimates that about 2000 new cases of severe precancerous abnormalities (40%), 142 new cervical cancer diagnoses (62%), and 55 deaths could be prevented each year if 80% of girls in Switzerland were vaccinated annually [11]. In light of these and similar statistics, many countries recommend HPV vaccination for adolescents. For example, in the US, the first author’s country of residence, HPV vaccination is recommended for boys and girls aged 11 and 12 [10]. In Switzerland, HPV vaccination is recommended for all 11- to 14-year-olds, and young people are considered able to benefit from vaccination until the age of 26 [9].

Even though the HPV vaccine can prevent cervical cancer, many parents are reluctant for their adolescents to be vaccinated. In the US, according to the Centers for Disease Control and Prevention, only 43% of adolescents are up to date on their HPV vaccination, as defined by having received both of the two recommended doses [12]. The estimated immunization rate of 16-year-old girls in Switzerland is 48% [13]. This is still unsatisfactorily low. Reasons for non-compliance vary, yet much non-compliance results from deliberate avoidance. Extensive research literature suggests that reasons for opposing vaccination in general include concerns about vaccine safety and efficacy and distrust of the conventional medical establishment and government as health information sources [14,15]. People also avoid vaccination against diseases that they perceive as not serious or eradicated in their areas. In the case of HPV vaccination, individuals often express concerns about the HPV vaccine being too new to have accumulated sufficient long-term safety data [16]. Parents are also concerned about the perceived connection between HPV vaccination and early sexual activity [16].

### 1.2. ‘Under the Hood’ of the Public’s Concerns about Vaccine Safety

Although we know that concerns about safety are the primary cause of refusing vaccination, very few pre-COVID-19 studies focus on understanding specific beliefs about safety and the knowledge that underlies them. Reviews of anti-vaccination websites over the span of a decade suggest that they endorse views that vaccines cause idiopathic diseases, include poisonous additives and ingredients, and erode the body’s natural immunity [17,18]. Thus, it is reasonable to assume that some individuals’ safety concerns are related to these views.

Increased attention to vaccine hesitancy during the COVID-19 pandemic highlighted that many misconceptions about safety have to do with vaccine biology (e.g., COVID-19 vaccines alter recipients’ DNA), as well as mistrust of governments, science authority, and healthcare establishment [19].

Gust and colleagues warn health professionals against the dichotomous categorization of parents into ‘vaccine supporters’ and ‘vaccine deniers’ [20]. They categorized parental vaccination attitudes into five clusters, ranging from ‘Immunization advocates’ (33% of respondents) to ‘fencesitters’ (13.2%) and ‘worried’ (2.6%). The authors showed that while only ‘fencesitters’ and ‘worried’ did not think that the benefits of immunization outweighed the risks, all groups had some safety questions and concerns. In a follow-up qualitative study, Gust and colleagues investigated concerns, information needs, and information preferences of ‘fencesitters’ and ‘worried’ mothers [21]. They found that these mothers had many specific safety questions and were interested in receiving tailored information. Examples of information needs included wanting to know what was inside vaccines, the relationship between vaccination and autism, and whether it was possible to ‘overload’ a child’s immune system.

In the aforementioned qualitative study, Zeyer and Sidler [4] interviewed Swiss student teachers (themselves within the recommended age for HPV vaccination) about their HPV vaccination concerns. The authors discovered a misconception about HPV vaccine safety that involved the belief that the HPV vaccine contains an attenuated (weakened) virus that may change back into a virulent form and cause HPV infection. This misconception was linked to not knowing that the HPV vaccine is genetically engineered and contains only synthetic markers of HPV that are capable of triggering immune response but do not possess any other functions of the actual virus.

### 1.3. Depth of Vaccine Safety Information Communicated to the Public

Gust and colleagues [20] and Zeyer and Sidler [4] explain that addressing individuals’ safety concerns on a conceptual level requires bringing in a certain depth of biological information (e.g., referring to structure and function of viruses, mechanism of an antigen’s interaction with a cell, etc.). Most laypeople do not have sufficient background knowledge to engage in such discussion easily. Equally, most pamphlets, websites, and brochures do not attempt such a level of complexity. Instead, they address the risks and safety by quoting specialist public health organizations (e.g., WHO, CDC) and by providing vaccination safety statistics. Public health brochures and websites typically discuss vaccination using factual statements about vaccination safety, effectiveness, and benefits, and provide practical vaccination information (e.g., places to receive vaccination). These documents do not introduce complex biological concepts that may be essential in addressing safety information needs and dismantling misunderstandings around vaccination.

### 1.4. Vaccination-Related Biology Education in School Science

One place where individuals encounter information about complex biological mechanisms pertaining to cells and viruses is the school science classroom. However, science education systems in most countries do not include in-depth coverage of microbiology and immunology that would form the conceptual basis for understanding vaccination [22,23]. Studies suggest that European and US students at all grade levels have a limited understanding of viruses, contagion, vaccination, and vaccine-preventable diseases. For example, in a study with a sample of 11-year-olds in the UK, Byrne and Grace [22] found that while most participants knew that microorganisms could cause diseases, their understanding of vaccination-induced prevention was very limited. Many thought that vaccines attacked and killed pathogens, thus essentially viewing vaccines as medicine. While these students were young, other studies suggest that misconceptions about microorganisms, infection, and vaccination persist into later school years. Focusing on knowledge about influenza, Romine, Barrow, and Folk [22] discovered that Midwestern US high school students (grades 9–12) hold a number of misconceptions about vaccines and vaccination, including the belief that vaccines act as medicine. In a cross-sectional study in Austria, Simon et al. (2017) found that seventh graders, tenth graders, and first-year university students held serious misconceptions about the biology of viruses that was likely to impact their understanding of vaccination.

Another common misconception found among adolescents [24] and young adults [4] is the belief that vaccines cause diseases they are designed to prevent. Adolescents and young adults also have a limited understanding of the structure and life cycle of microorganisms and cannot distinguish between viruses and bacteria [25,26,27,28]. While most of the studies into students’ and young adults’ understanding of microbiology and immunology aim to characterize misconceptions and develop assessment instruments, some include interventions that aim to increase understanding. For example, Keselman, Kaufman, Kramer, and Patel [29] showed that integrating units about viruses and the immune system reduced adolescents’ misconceptions about HIV. Dumais and Hasni [27] found that a curriculum highlighting viral structure and function improved Canadian students’ understanding of influenza biology.

### 1.5. Study Rationale and Research Questions

Decisions about how to decrease misconceptions about HPV vaccination and increase vaccination rates must take into account not only what information to provide, but also in what contexts and situations to provide information. Should this be carried out in the science classroom, the health classroom, or through out-of-school public health channels? At the moment, the wall between health communication and science education is difficult to scale on both sides [30]. On the science education end, despite the general belief that science learning is good for all students and not just future scientists, few studies have attempted to connect biological knowledge with reasoning and decision making in the context of health and disease [30,31]. In the world of classroom practice, science learning objectives pertaining to viruses and cell biology do not aim to influence vaccination behavior. On the health communication end, information brochures and pamphlets are often disconnected from the things people worry about. For example, Hall, Howard, and McCaffery [32] found that patient information leaflets are often inadequate in answering women’s questions about HPV.

At present, information about HPV and HPV vaccination is most likely to be communicated to young people and their parents outside of school through public health brochures and websites. Little research has been conducted on the impact of HPV brochures on safety beliefs and vaccination attitudes. The present study aims to answer two questions. First, it quantitatively examines whether a standard public health brochure affects student teachers’ perception of the effectiveness, safety, and importance of HPV vaccination. Second, it investigates whether supplementing a traditional HPV information brochure with text about relevant biological concepts is more effective than the brochure alone in communicating the safety of the vaccine. In particular, the study investigates the impact on beliefs of a biological explanation about the mechanism by which HPV vaccination acts on the immune system. It focuses on beliefs about vaccination importance, safety, effectiveness, and, specifically, the possibility of the vaccine causing HPV infection. While the study is exploratory, we hypothesize that the biology-enhanced text will have a greater positive effect on safety beliefs than the traditional brochure. The study aims to generate questions that will set a research agenda for further examining the relationship between health and science education and between formal education and public health communication.

## 2. Methods

### 2.1. Participants and Setting

Participants in this study were enrolled students from the Lucerne University of Teacher Education in Switzerland. They were all enrolled in a course titled ‘Health and Illness in School’ in the autumn of 2016 taught by the second author. The aim of the course was to prepare students, future Swiss school teachers, for promoting health and addressing health concerns among Swiss school children. The course enrollment included 350 students. Three hundred and eleven students chose to participate in the study. Of these, 232 completed pretest and posttest questionnaires and were included in the analysis (130 in the standard brochure and 102 in the extended brochure condition). Twenty-nine percent of the participants were male and seventy percent were female. Ninety-five percent were between twenty and twenty-eight years old. The majority (70%) were training to be primary school teachers.

The study focused on future teachers both because most of them were within the age range recommended for HPV vaccination and because they would soon be in a position to influence the health decisions of others. When it comes to addressing HPV vaccination in the classroom, this may be a concern because student teachers’ understanding of antibodies and immunity is not free from misconceptions [24]. 

### 2.2. Procedure

The study protocol was exempt from IRB review by the Office of Human Subjects Research of the US National Institutes of Health. Participants provided informed consent in writing. A pretest–posttest design was used in this study. At the outset, participants completed a questionnaire where they were asked to rate ten statements (see Box 1) on a 5-point scale, using the following scale: 1 = ‘strongly disagree’, 2 = ‘somewhat disagree’, 3 = ‘neither agree, nor disagree’, 4 = ‘somewhat agree’, and 5 = ‘strongly agree’. To establish construct validity, the questionnaire, grounded in the Health Belief Model (HBM) of health behavior [33], was based on the authors’ previous work [34]. This work drew from a review of studies on vaccine beliefs [35]. For this study, our pretest questionnaire was expanded to address concerns about side effects included in CDC consumer health materials (e.g., https://www.cdc.gov/hpv/parents/questions-answers.html (accessed on 12 January 2022). Based on the literature review and our prior findings of concerns related to misconceptions around vaccination-related risks, the primary focus of our questionnaire was on beliefs about vaccination safety. To establish content validity of the questionnaire, two health professionals—a physician and a nurse—were asked to map each of the ten statements in the questionnaire to one of three relevant concepts of the HBM (i.e., perceived susceptibility to cervical cancer, perceived benefits of vaccination, and perceived costs/risks of vaccination) or to mark the statement as the overall summative statement. The health professionals mapped the statements with 100% agreement. In addition, when asked to add possible additional safety concerns that someone may have about HPV vaccination, both judged the coverage of statements 5 to 9 as comprehensive. The pretest questionnaire also included demographic questions about the participants’ age, gender, and educational focus.

Box 1 **Questionnaire statements.**

1. Without HPV vaccination, a woman may get cervical cancer.

2. HPV vaccination is important.

3. HPV vaccination is safe.

4. HPV vaccination is effective against cervical cancer.

5. Serious side effects can occur with HPV vaccination.

6. In some cases, HPV vaccine may cause HPV infection.

7. In some cases, HPV vaccination may cause cervical cancer.

8. In some cases, HPV vaccination may cause fertility problems.

9. In some cases, HPV vaccination may weaken the recipient’s immune system.

10. The benefits of HPV vaccination outweigh the risks of HPV vaccines.

After the pretest, participants were given a brochure about HPV vaccination to read. They read either the standard or extended version of the brochure, which were both developed using a US CDC brochure (see https://www.cdc.gov/std/hpv/stdfact-hpv-vaccine-young-women.htm (accessed on 12 January 2022). The CDC brochure was selected as an authoritative document that could provide a basis for further cross-cultural comparisons. The brochure was shortened and edited to focus on the information pertaining to the questions in Box 1. As the study was conducted in Switzerland, the authors also removed the information pertaining to the US healthcare system (e.g., how to pay for vaccination). The resulting brochure included the following sections: *What is HPV?; What is HPV vaccination?; How effective are the HPV Vaccines?; Are HPV vaccines safe?* The information in these sections was presented in a clear factual manner, providing references to authoritative sources and statistics, but biological mechanisms were not described (e.g., ‘The vaccines were studied in thousands of people around the world, and these studies showed no serious safety concerns’).

In addition, the *extended* version of the brochure was modified by the text in Box 2. It included an in-depth biological explanation of the nature of viruses and the HPV vaccine, as well as the mechanism by which the HPV vaccine interacts with the immune system.

Participants were assigned to one of the two text conditions through a pseudo-random procedure: The proctor approached students in the order in which they sat in the class, alternatively handing out materials with the standard and the extended text versions.

Box 2 **Text added to the extended version.**

*1.1 What, exactly, is a virus (like HPV)?*

*Viruses, like HPV, are single-cell sub-microscopic organisms that can infect humans. As organisms, viruses are simpler than most living cells. They lack many of the parts (organelles) that cells usually have. Viruses can be thought of as containers with DNA that contains instructions for building new viruses. Like all living things, viruses can reproduce, making more of themselves. But viruses are so simple that they so can only reproduce by entering a host cell. This is where our trouble begins. Viruses infect (enter) host cells, take over their reproductive machinery to make more viruses, and damage them in the process. That’s how viral infections make us ill.*

*2.2 How does HPV vaccination protect against HPV?*

*Vaccination makes a person resistant to an infection by teaching the immune system to recognize it and trigger a response. The immune system is the body’s system for destroying intruders, such as viruses. This system works partly by producing proteins called “antibodies” that connect to the intruders’ proteins called “antigens.” These antigen-antibody complexes signal other mechanisms of the immune system to destroy the intruder. Different viruses have different antigens that require different specialized antibodies. Because of this, the immune system is often more effective in responding to previously encountered antigens. Vaccines contain antigens, giving the immune system the opportunity to learn to recognize viruses and other infectious agents. Vaccines can introduce antigens as part of weakened viruses (never fully strong viruses), dead viruses, or through genetically engineering of harmless viral antigens. The HPV vaccine contains only antigen molecules of HPV. These molecules can stimulate the immune system to produce HPV antibodies, but cannot enter host cells, and thus can’t use their reproductive machinery and damage them. Therefore, HPV vaccination can never cause HPV infection. It can, however, prevent HPV infections which cause cervical cancer.*

After completing the pretest and reading their texts, students handed these materials to the proctor. The posttest, taken at the end of the class, approximately one hour after reading the brochures, was identical to the pretest, with the exception of the demographic questions.

All study materials were originally created in English and later translated into German by the second author, who is fluent in both languages. An independent bilingual speaker verified the translation’s accuracy by back-translating materials into English and comparing translations with the original.

## 3. Data Analysis

The analysis was conducted for the following dependent variables, which were developed to reflect theoretical HBM components on the basis of students’ responses:

1. **Susceptibility to HPV Infection (Susceptibility).** This single-item variable, ranging in value from 1–5, reflected the rating of the statement ‘Without HPV vaccination, a woman may get cervical cancer’.
2. **Importance of HPV Vaccination (Importance).** This two-item variable, ranging in value from 2–10, reflected the sum of ratings of the following two statements: ‘HPV vaccination is important’, and ‘The benefits of HPV vaccination outweigh the risks of HPV vaccines’. As measured by Crohnbach’s alpha, the internal consistency of the importance variable was α = 0.636 in the pretest and α = 0.786 in the posttest.
3. **Safety of HPV Vaccination (Safety).** This 6-item variable, ranging in value from 6–30 reflected the sum of the ratings of the ‘HPV vaccination is safe’ statement and inverse ratings of the following five statements: ‘Serious safe effects can occur with HPV vaccination’, ‘In some cases, HPV vaccine may cause HPV infection’, ‘In some cases, HPV vaccination may cause cervical cancer’, ‘In some cases, HPV vaccination may cause fertility problems’, and ‘In some cases, HPV vaccination may weaken the recipient’s immune system’. As measured by Cronbach’s alpha, the internal consistency of the safety variable was α = 0.717 in the pretest and α = 0.864 in the posttest.
4. **Effectiveness of HPV Vaccination (Effectiveness).** This single-item variable, ranging in value from 1–5, reflected the rating of the statement ‘HPV vaccination is effective against cervical cancer’.

The data were analyzed through repeated-measures MANOVA and repeated-measures ANOVA, with time elapsed between the pretest and posttest as the within-subject factor and type of text as the between-subject factor.

## 4. Results

Statistical tests are two-tailed, unless otherwise specified.

### 4.1. Sample-Wide Comparisons

Repeated-measures MANOVA was used to analyze the impact of the procedure on the four HBM factors. The multivariate analysis produced an overall highly significant result of time (F(4, 227) = 2783.26, *p* < 0.001), but no significant effect of time by text type interaction. Similarly, univariate analysis of each factor produced a significant time effect and no significant interaction effect (see Table 1).

In addition, repeated-measures ANOVA was performed on participants’ rating of the ‘In some cases, HPV vaccination may cause HPV infection’ statement, as this statement was the most directly targeted by the biological text of the extended version. Similar to the overall safety factor effects, there was a significant effect of time, but not of time by text interaction (Table 1).

### 4.2. Post Hoc Restricted Range Sample Comparisons

After obtaining the whole-sample results, we further hypothesized that participants with different initial beliefs about HPV vaccination safety might be affected differently by the biology in the extended text. Specifically, we hypothesized that participants who viewed vaccination as very safe at the outset may be close to the ceiling level and not demonstrate much of a change in their safety perceptions scores, while participants with a very high level of initial skepticism may have their beliefs grounded in a complexity of factors not easily affected by a single biological passage. Participants with moderate or uncertain initial views, on the other hand, may be most likely to change their views in response to a biological explanation.

To test this hypothesis, we divided the original sample into three groups based on participants’ pretest perceived safety scores. A participant answering ‘neither agree, nor disagree’ to all six safety questions would have attained a score of 18. That score (18), achieved by 57 of the 268 participants who provided complete data for the safety factor questions, also represented a very distinctive mode in our data set. The second most frequent score (of 16) was obtained by 23 participants. We, thus, defined the ‘initial moderate safety belief’ group as those whose pretest scores fell within +/−2 points of 18, ranging from 16 to 20. Participants with pretest safety scores of 15 or below were defined as the ‘initial low safety belief’ group, and participants with pretest safety scores of 21 or above were defined as the ‘initial high safety belief’ group. Notice that these are average scores. For example, participants with a score of 18 could agree or disagree with some of the six safety questions, as long as they had an average score of 3, i.e., a total score of 18, for all six questions.

The perceived safety of HPV vaccination was analyzed for each group using repeated-measures ANOVA with time as a within-subject factor and type of text as a between-subject factor. The results are presented in Table 2. For each of the three groups, the analysis revealed the overall significant effect of time, which remained significant when Bonferroni correction for multiple comparisons was applied. The effect of time by text type interaction, in a one-tail test with Bonferroni correction applied, was significant (F(1, 127) = 3.952, *p* = 0.025) for the *Initial Moderate Safety Belief Group* and not significant for the other two groups.

In order to calculate the size of this effect for the Initial Moderate Safety Belief Group, Cohen’s d for pretest–posttest-controlled studies [36] was applied. This yielded an effect size of d = 0.46, which, in a pedagogical setting, can be considered as a medium effect [37].

### 4.3. Note on Effect Size Computation

In this study, we chose to estimate the size of the intervention effect by calculating Cohen’s d [38] for pretest–posttest-controlled studies according to Lenhard and Lenhard [36]. These authors suggest calculating the effect size of the pre- and post-measurement using Hedges’ g [38] and then subtracting the two effect sizes from each other. In this way, different group sizes, as well as pretest differences, are corrected.

While Lenhard and Lenhard standardize both pretest and posttest standard deviations, another common approach standardizes pretest standard deviations only [39]. This type of calculation yields a much higher Cohen’s d of 1.17, which would signify a very large effect of our intervention.

Nevertheless, we prefer using Lenhard and Lenhard’s method, because scales are used in two different ways in our study. On the one hand, they provide a continuous measure in comparing pretest and posttest scores. On the other, they are used as an indicator of an underlying categorical variable. By selecting a subsample as described, the variance will necessarily be reduced for the pretest scores for the selected group and the calculation of effect size will be inflated. Overall, Cohen’s d calculated according to Lenhard and Lenhard will surely be on the safe side, and it can be expected that the substantial value of this variable will be rather larger than the value of 0.49 that we are using in our argumentation.

## 5. Discussion

This study examined the impact of a standard public health brochure on young adults’ perception of the effectiveness, safety, and importance of HPV vaccination. Furthermore, it also investigated the added effect of supplementing the standard brochure with a biological explanation of vaccine-induced immunity.

Participants in both conditions showed a significant positive change in their beliefs about vaccination safety, effectiveness, and importance in preventing cervical cancer. These results agree with Zeyer and Knierim’s [2] quantitative study in which participants’ general beliefs about vaccination were positively influenced by an official video produced by the Swiss Paediatric Society about tetanus. However, although participants’ views of vaccination safety grew between the pretest and posttest, the average posttest safety belief score of 23.97 out of 30 suggests there is still much worry and ambivalence about HPV vaccination. The study did not ask participants about their vaccination status and their actual intention to obtain HPV vaccination and did not attempt to relate the level of attitude change to behavior. However, it can be assumed that the HPV vaccination status of the participants reflects the general situation in Switzerland (see above, section “Human Papillomavirus (HPV): Epidemiology, Impact, and Prevention”).

Contrary to our hypothesis, adding biology-enhanced text to the standard brochure did not have an overall positive effect on participants’ beliefs about HPV vaccination safety. However, post hoc analysis showed a significant gain in safety beliefs for the biology-enhanced text in the subgroup comprised of *fencesitters*, i.e., participants whose pretest safety beliefs were moderate, rather than high or low. Although the overall impact is limited, a single read of a standard brochure may positively impact some individuals’ beliefs about HPV vaccination. Furthermore, the biological explanation had an additional positive impact on the subgroup of those concerned about vaccine safety whose initial negative attitudes were not too strong. Further research should explore the potential of in-depth science-based explanations, as well as the best strategies and contexts for delivering them.

When it comes to addressing the role of conceptual science knowledge, the study reveals several possibilities that merit further investigation. It presents an interesting finding, though highly exploratory in nature, about the impact of a biological explanation on participants’ beliefs about the safety of HPV. While there was no overall effect of enhancing the brochure with biological explanations, such an in-depth explanation obviously may have played a role for *fencesitters*, i.e., those whose initial beliefs about HPV vaccination safety were neutral. This finding is strengthened through the study’s use of a restricted data range and by a considerably high effect size of the intervention. While beyond the scope of the present study, further research may illuminate how individuals with different initial perceptions of vaccination and different levels of certainty respond to biological explanations. It also suggests that focusing educational and explanatory efforts on fencesitters may have positive practical implications and should be explored in other contexts, such as promoting COVID-19 vaccination.

With regard to seeking out a larger and more definitive impact of biological knowledge, it appears that a couple of printed paragraphs of biological explanation presented in isolation from the rest of relevant biology did not affect participants’ beliefs over and above the factual text. More research, with a qualitative focus, is needed to determine why this was the case. One limitation of the current design was its inability to assess text comprehension. It is possible that the readers understood the text but found it unconvincing or that it did not address all of their concerns about vaccination safety. Vaccine hesitancy is a complex phenomenon that includes many cognitive and non-cognitive components. Most effective public health programs for addressing vaccine hesitancy intervene on multiple levels (individual, family, community), involve multiple components and materials, and unfold over a period of time [40]. It is also possible that the brochure format was not sufficient for bridging gaps in conceptual understanding.

## 6. Conclusions

We often vainly search for spectacular interactions between science education and health behavior, while missing the more delicate, but no less important, benefits on a deeper level. This may be the significance of this study, even if, at a first glance, one might be disappointed with the findings. In fact, important biomedical information on HPV vaccination does not result in dramatic changes in beliefs among students. One might even be tempted to discard the intervention tested in this study.

However, this might be an important conceptual mistake. Rather, vaccination hesitancy is a dynamic and challenging period of indecision around whether or not to accept vaccination [40]. The spectrum of hesitancy is wide and varying, ranging from ‘advocates,’ who do not question vaccination at all, to the ‘worrieds,’ who reject outright vaccination [41]. While ‘accepters’ require no convincing about vaccination, the ‘rejecters’ do not respond to any argument in support of vaccination.

Yet, and this seems to be suggested by our results, for the ‘moderates,’ who lie in-between the accepters and rejecters, information about the biomedical safety of HPV vaccination may be just the right nudge towards a health literate decision. It may be in this unspectacular way that scientific literacy supports health decisions in everyday life and that the science classroom can become an important setting for health education. Today, individuals are expected to be active participants in their healthcare, collaborating with health professionals in choosing the course of action in care, treatment, and prevention. Participatory medicine encourages patients to ask questions and to engage in shared decision-making [30]. Our study suggests that in the process of improving participation, scientific literacy, though it may not be the “silver bullet” for health literate decisions, can well be an important piece to the puzzle. In particular, this realization may reshape the focus of research interests on the moderate and fine grained. Through this type of educational research, the effects of science education on students’ (and citizens’) health literacy may be better observed than on the macro-level of spectacular life and death decisions, which are usually the focus of interest [42].

This study looked at public health texts, which were translated from English to German. They were enhanced with additional information that had been identified as useful in a local Swiss study. Targeted health communication is and will remain an important mission of public health. Long, interactive discussions with patients and consumers are not feasible in every setting where health information delivery takes place. Consequently, health information brochures and handouts are and will always be important tools in delivering health information. At the same time, this study makes a case for fostering alliances between public health and school science education and for bringing health topics into science education. While the impact of conceptual knowledge on daily life needs to be further explored, it is certainly reasonable to assume that it is more likely to be impactful when delivered in a connected, comprehensive, expertly facilitated characteristic of the science classroom [27,30,43]. Additionally, topics of microbiology and immunology that are essential to understanding HPV vaccination are also very relevant for teaching other essential public health, science, and society concerns. Consequently, public health will also benefit from constituents with a sound conceptual base for dealing with health information on a variety of complex topics, from antibiotic resistance to pandemics and herd immunity [23,25]. HPV biology education would also be incomplete without general cancer biology. Topics that pertain to cancer are sensitive and addressing them to school-aged children and teens should be carried out with the utmost care. Yet, there is some indication that science teachers think that these topics are important and belong in the science classroom and that the emotional aspects are manageable [44].

Lastly, in the COVID-19 era, when vaccination is hotly debated and often doubted by the public of many countries, it is important to remember that when it comes to scientific literacy, conceptual knowledge is only one component. Individuals’ scientific knowledge and their attitudes towards science and scientists play a very important role in building trust and acceptance of vaccines, especially in a context in which health becomes politicized [45]. Therefore, placing an emphasis on how scientists generate and validate their findings, as well as facilitating dialogue between scientists and the public becomes very important [46].

## Figures and Tables

**Table 1:** Sample-wide effects of reading the texts on perception of HPV vaccination.

	Pretest M(SD)	Posttest M(SD)	N	F Time *	*p*
	**Perceived Susceptibility to HPV**				
Standard text group	3.90 (1.08)	4.48 (0.89)	132		
Extended text group	3.82 (1.33)	4.50 (0.90)	103		
Total	3.86 (1.19)	4.49 (0.89)	235	66.34	<0.001
	**Perceived Importance of HPV Vaccination**				
Standard text group	6.67 (1.88)	7.86 (1.98)	132		
Extended text group	6.82 (1.94)	8.37 (1.69)	103		
Total	6.74 (1.90)	8.09 (1.87)	235	149.35	<0.001
	**Perceived Safety of HPV Vaccination**				
Standard text group	17.29 (4.07)	23.34 (5.35)	132		
Extended text group	18.07 (3.48)	24.77 (4.86)	103		
Total	17.63 (3.83)	23.97 (5.17)	235	393.32	<0.001
	**Perceived Effectiveness of HPV Vaccination**				
Standard text group	3.62 (0.89)	4.28 (0.93)	132		
Extended text group	3.55 (0.98)	4.03 (1.24)	103		
Total	3.59 (0.93)	4.17 (1.08)	235	55.92	<0.001
	**Perceived Possibility of HPV Vaccine Causing HPV Infection**				
Standard text group	3.32 (0.94)	2.10 (1.21)	143		
Extended text group	2.99 (1.00)	1.69 (1.04)	112		
Total	3.18 (0.98)	1.92 (1.16)	255	212.075	<0.001

*— degrees of freedom (1, 230).

**Table 2:** Post-reading perception of HPV vaccination safety for groups with different initial beliefs.

	Pretest M(SD)	Posttest M(SD)	N	F Time, df	*p*
	Initial Low Safety Belief Group				
Standard text group	12.53 (2.28)	20.86 (5.95)	43		
Extended text group	13.41 (1.65)	21.68 (5.71)	22		
Total	12.83 (2.12)	21.14 (5.34)	65	135.971 (1.63)	<0.001
	Initial Moderate Safety Belief Group				
Standard text group	18.01 (1.28)	23.83 (4.43)	70		
Extended text group	17.98 (1.21)	25.27 (4.42)	59		
Total	18.00 (1.24)	24.49 (4.47)	129	312.356 (1.127)	<0.001
	Initial High Safety Belief Group				
Standard text group	23.00 (2.18)	25.68 (4.82)	25		
Extended text group	22.87 (1.87)	26.39 (4.05)	23		
Total	22.94 (2.01)	26.02 (4.44)	48	24.610 (1.46)	<0.001

## Data Availability

The data presented in this study are available on request from the corresponding author. The data are not publicly available due to privacy restrictions.
